# A context-aware temporal modeling through unified multi-scale temporal encoding and hierarchical sequence learning for single-channel EEG sleep staging

**DOI:** 10.1371/journal.pone.0358241

**Published:** 2026-09-30

**Authors:** Amir Ali Vakili, Salar Jahanshiri, Armin Salimi-Badr

**Affiliations:** Faculty of Computer Science and Engineering, Shahid Beheshti University, Tehran, Iran; Institute of Wenzhou, Zhejiang University, CHINA

## Abstract

Automatic sleep staging is a critical task in healthcare due to the global prevalence of sleep disorders. This study focuses on single-channel electroencephalography (EEG), a practical and widely available signal for automatic sleep staging. Existing approaches face challenges such as class imbalance, limited receptive-field modeling, and insufficient interpretability. This work proposes TempoSleep, a context-aware framework for single-channel EEG sleep staging, with particular emphasis on improving detection of the N1 stage. Many prior models operate as black boxes with stacked layers, lacking clearly defined and interpretable feature extraction roles. TempoSleep combines compact multi-scale feature extraction with temporal modeling to capture both local and long-range dependencies. To address data imbalance, especially in the N1 stage, class-weighted loss functions and data augmentation are applied. EEG signals are segmented into sub-epoch chunks, and final predictions are obtained by averaging softmax probabilities across chunks, enhancing contextual representation and robustness. The proposed framework achieves an overall accuracy of 89.72% and a macro-average F1-score of 85.46%. Notably, it attains an F1-score of 61.7% for the challenging N1 stage, demonstrating a substantial improvement over previous methods on the SleepEDF datasets.These results indicate that the proposed approach effectively improves sleep staging performance while providing insights into its prediction behavior and supporting its potential application in automatic sleep staging.

## Introduction

Artificial intelligence (AI) has become a transformative tool in modern disease diagnosis, enabling automated, data-driven analysis across a wide range of medical applications, including medical imaging, biomedical signal processing, and clinical decision support systems [1,2]. By learning complex patterns from large-scale and high-dimensional healthcare data, AI-based methods have demonstrated the potential to improve diagnostic accuracy, reduce clinician workload, and enable early detection of diseases. In particular, deep learning techniques have shown remarkable success in modeling temporal and spatial dependencies in physiological signals, making them well suited for tasks involving continuous health monitoring [3–5].

Sleep plays an important role in maintaining and regulating the body’s biological functions at the molecular level. Proper sleep restores human physical and mental health and helps ensure optimal performance throughout the day [6,7]. Sleep is divided into three main types: non-rapid eye movement sleep (NREM), rapid eye movement sleep (REM), and the wake state. NREM sleep itself includes four stages: N1, N2, N3 and N4, with the last stage transitioning into REM sleep [8]. These two types of sleep alternate cyclically during the sleep process, and if this cycle is disrupted or certain stages are skipped, sleep quality is impaired. Accurate classification of the N1 stage holds distinct clinical significance. As the primary transitional phase between wakefulness and deeper sleep, N1 is highly sensitive to sleep fragmentation, sleep-onset insomnia, and frequent arousals. Misclassification or underestimation of N1 can mask underlying pathologies. Recent studies emphasize the critical need for precise N1 monitoring when evaluating epileptic seizure occurrences and related sleep disruptions [9], as well as in understanding sleep’s multidimensionality and its role in cognitive transitions [10]. Despite its clinical importance, N1 remains notoriously difficult to classify automatically due to its low-amplitude, mixed-frequency characteristics and its signal resemblance to wakefulness and REM stages.

In today’s world, the assessment of sleep stages is mainly performed manually by specialists in this field, which comes with limitations. Sleep experts classify sleep stages using hypnograms; however, they may struggle to accurately detect slow changes in brain signals or interpret complex rules. Furthermore, differences of opinion among experts can reduce accuracy [11,12].

Other challenges also exist, such as patient comfort, diagnostic costs, and the use of portable and wearable devices. To address these issues, we need a fully automated and low-cost method for performing this task. While multimodal polysomnography (combining EEG, EOG, and EMG) remains the gold standard for clinical sleep staging due to its comprehensive physiological monitoring, it requires a highly specialized clinical environment. Although the use of additional modalities increases the number of physiological signal dimensions and provides richer information for the model, it does not increase the number of independent patients or subjects. Moreover, acquiring EOG and EMG signals in addition to EEG requires more sensors and a more complex recording setup, which can increase cost, reduce patient comfort, and limit scalability for home-based or wearable monitoring. In contrast, single-channel EEG approaches offer substantial strengths: they are highly cost-effective, maximize patient comfort, and facilitate longitudinal, at-home ambulatory monitoring through wearable devices. By relying on a single sensor, these methods significantly lower the barrier to continuous automated clinical deployment, making the extraction of rich temporal features from a single channel a critical area of algorithmic development.

Early attempts at automation were carried out using classical machine learning algorithms. Those approaches relied heavily on handcrafted features extracted from different physiological signals such as EEG, EOG, and EMG [13–15] and other signals like heart rate and actigraphy [16]. Typical classifiers used in these studies include SVM, Random Forest, K-NN, Decision Trees, and Ensemble Methods. For example, Willemen et al. [17] employed statistical features alongside an SVM classifier, reaching 69% accuracy. Research has also focused on developing efficient methods using a single sensor to enhance practicality. Dimitriadis et al. [18] proposed a single-EEG-sensor technique based on the dynamic reconfiguration of cross-frequency coupling (CFC) using a Naive Bayes classifier. Similarly, Zhu et al. [19] introduced a novel approach by mapping single-channel EEG signals into difference visibility graphs (DVG) to extract graph-based features, which were then classified with an SVM, achieving an accuracy of 87.5% for six sleep stages.

As a replacement for classical methods, deep learning models have been used to switch from handcrafted features to representation learning. Recent studies have further demonstrated the effectiveness of CNN-based and hybrid deep learning architectures for EEG signal analysis and sleep-stage recognition. Wang et al. proposed a multi-modal framework with contrastive learning and sequential encoding for enhanced sleep-stage detection, showing the benefit of combining representation learning with temporal sequence modeling [20]. In related EEG applications, Wang et al. introduced a Multi-Scale-CNN for P300 detection, demonstrating that convolutional kernels at different temporal scales can improve EEG pattern recognition [21]. Similarly, Wang et al. linked an attention-based multi-scale CNN with a dynamical graph convolutional network for EEG-based driving fatigue detection, highlighting the usefulness of attention mechanisms and graph-based temporal-spatial modeling [22]. Ji et al. proposed a subject-specific CNN model with parameter-based transfer learning for SSVEP detection, showing the relevance of transfer learning in adapting CNN-based EEG models to individual subjects [23]. In addition, Ji et al. developed CBAM-DeepConvNet for asymmetric visual evoked potential recognition, further demonstrating the effectiveness of combining convolutional neural networks with attention modules for EEG decoding [24]. These studies support the broader applicability of CNN-based, multi-scale, and attention-enhanced architectures for extracting discriminative information from EEG signals, motivating their use in automatic sleep-stage classification. Eldele et al. [25] proposed a novel attention-based deep learning architecture called AttnSleep to classify sleep stages using single-channel EEG signals. Their model begins with a feature extraction module based on a multi-resolution convolutional neural network (MRCNN) and adaptive feature recalibration (AFR), followed by a temporal context encoder (TCE) that leverages a multi-head attention mechanism with causal convolutions to capture temporal dependencies. Evaluations on three public datasets demonstrated that AttnSleep outperformed state-of-the-art techniques in terms of different evaluation metrics. Another method was proposed by Supratak et al. [26], who developed DeepSleepNet, a deep learning model for automatic sleep stage scoring based on raw single-channel EEG. The model combines convolutional neural networks for time-invariant feature extraction and bidirectional long short-term memory (BiLSTM) networks to learn transition rules among sleep stages. Tested on multiple datasets with different sampling rates and scoring standards, DeepSleepNet achieved overall accuracies of 86.2% (MASS) and 82.0% (SleepEDF). Phan et al. [27] introduced SeqSleepNet, a hierarchical recurrent neural network designed to perform sequence-to-sequence sleep staging. At the epoch level, the network employs a filterbank layer to learn frequency-domain filters and an attention-based recurrent layer for short-term sequential modeling, while at the sequence level, another recurrent layer models long-term dependencies across multiple epochs. Their method achieved an overall accuracy of 87.1% and a macro F1-score of 83.3% on a large public dataset. Mousavi et al. [28] presented SleepEEGNet, a sequence-to-sequence deep learning approach using single-channel EEG signals. The model integrates deep convolutional neural networks to extract temporal and frequency features with a recurrent architecture to capture long-term dependencies. To mitigate class imbalance, novel loss functions were applied to balance misclassification errors across stages. On the SleepEDF dataset, SleepEEGNet achieved 84.26% overall accuracy and a macro F1-score of 79.66%, outperforming many existing methods.

The main problem in this field is the lack of samples in the N1 class, and N1 samples are mainly misclassified as Wake or N2. Different methods have been used to resolve this issue. Cheng [29] used generative adversarial networks to augment data and increase the number of samples for minority classes, achieving an accuracy of 86.8%. Many other researchers used weighted cross-entropy to tackle this problem. Although this approach increases accuracy in the N1 class, it often leads to lower accuracy in other classes, making it an inappropriate solution. Although the above deep learning approaches have achieved substantial progress in automatic sleep staging, the limitations of existing CNN-LSTM and related architectures can be summarized from several perspectives. Many CNN-LSTM-based methods, such as DeepSleepNet and SleepEEGNet, use convolutional layers as general feature extractors followed by recurrent layers for temporal modeling. While this strategy is effective, it may increase computational cost when recurrent modules process long raw sequences or high-dimensional feature maps. Sequence-to-sequence models such as SeqSleepNet and XSleepNet improve temporal dependency modeling, but often rely on more complex input representations or hierarchical processing. Attention-based and multi-scale models such as AttnSleep and SleepFocalNet improve feature extraction by emphasizing salient temporal patterns, but the feature extraction process is not always explicitly separated into interpretable computational stages such as local multi-scale extraction, temporal compression, and sequence-level modeling. Graph-based approaches such as SleepGCN explicitly model sleep-stage transitions, but require graph construction and introduce additional computational complexity. Data-augmentation-based approaches such as SleepEGAN attempt to address class imbalance, but generative augmentation can increase training complexity and does not fully resolve the intrinsic ambiguity of the N1 stage. These limitations motivate the proposed architecture, which separates the pipeline into multi-scale temporal feature extraction, temporal compression, hierarchical sequence modeling, and final classification. In particular, depthwise separable convolutions reduce convolutional parameter growth, while temporal compression reduces the sequence length passed to the BiLSTM layers from 500 raw samples to 5 latent time steps. Therefore, the proposed framework is designed to reduce computational burden while preserving local and long-range temporal information relevant to sleep-stage classification, especially for the challenging N1 stage.

In this paper, we address these challenges through a context-aware preprocessing strategy and a novel unified architecture. Our main contributions are:

- A novel preprocessing strategy with sub-epoch segmentation and voting to address limited receptive-field modeling by enhancing context and increasing training samples.
- A custom CNN architecture with multi-scale kernels and squeeze-and-excitation blocks for interpretable feature extraction with defined layer roles, enabling channel attention and temporal compression.
- A hierarchical BiLSTM with attention pooling to capture both short-term and long-term temporal dependencies, addressing the need for better temporal modeling.
- Comprehensive data augmentation strategies and class-weighted loss functions to mitigate the severe class imbalance problem, particularly for the challenging N1 stage.

The remainder of this paper is organized as follows. The Materials and methods section describes the proposed TempoSleep methodology, including data preprocessing, temporal feature extraction, and sequence modeling. The Results section presents the experimental setup and results, including comparisons with prior methods and ablation studies. The Discussion section discusses the implications of the findings, and the Conclusion section summarizes the paper.

## Materials and methods

In this section, we first explain the problem along with the underlying dataset. To enrich our training samples and improve the generalization of the proposed method, some data-augmentation processes are applied, too. Next, we explain the TempoSleep in detail.

### Data

In this study, we evaluated our method using two benchmark datasets from the SleepEDF Database Expanded: SleepEDF-20 and SleepEDF-78. SleepEDF-20 consists of recordings of 20 subjects between 25 and 34 years of age, with a total of 39 recordings. Each PSG record contains multiple channels, including EEG (Fpz–Cz and Pz–Oz), horizontal EOG (ROC–LOC), and chin EMG, sampled at 100 Hz. Only the Fpz–Cz EEG channel was used in this study, consistent with the objective of developing a lightweight and practical sleep staging framework based on minimal signal acquisition. Following the Rechtschaffen and Kales (R&K) standard, recordings were labeled as Wake (W), stages N1–N4, rapid eye movement (REM), Movement, or Unknown [30]. According to common and standard practice, we excluded the Movement and Unknown epochs and merged N3 and N4 into a single N3 stage, producing the standard five-class classification: [Wake (W), N1, N2, N3, REM].

SleepEDF-78 is a later expanded version of SleepEDF. It includes 197 polysomnography (PSG) recordings from 78 healthy participants (34 male and 44 female) aged between 25 and 101 years. For most subjects, two consecutive day–night recordings were available, except for three cases (subjects 13, 36, and 52). Similar to SleepEDF-20, PSG records in SleepEDF-78 contain EEG (Fpz–Cz and Pz–Oz), horizontal EOG (ROC–LOC), and chin EMG signals, sampled at 100 Hz and annotated with hypnograms in 30-second epochs by expert specialists. A summary of the dataset statistics is provided in Table 1.

**Table 1:** Summary of employed datasets.

Dataset	EEG channel	Epoch counts and proportion					
		Wake	N1	N2	N3	REM	Total
SleepEDF-20	Fpz–Cz	8285 (19.6%)	2804 (6.6%)	17799 (42.1%)	5703 (13.5%)	7717 (18.2%)	42308
SleepEDF-78	Fpz–Cz	69824 (35.0%)	21522 (10.8%)	69132 (34.7%)	13039 (6.5%)	25835 (13.0%)	199352

Various augmentation methods were applied to increase model stability and generalization, as well as mitigating overfitting problem. Each method was applied stochastically to simulate realistic variability and artifacts. The methods included:

- Additive Gaussian Noise: For simulating electrode and environmental noise [31].
- Temporal Scaling and Shifting: randomly compressing and stretching the signal to simulate physiological variability.
- Temporal Masking: zeroing out short intervals to force the model to exploit contextual information. This technique is also used to augment minority class samples.

### Proposed method

Fig 1 presents an overview of the proposed pipeline. The methodology consists of four main stages: (1) data preprocessing with sub-epoch segmentation and context window construction, (2) temporal feature extraction via a custom CNN with multi-scale kernels and squeeze-and-excitation blocks, (3) hierarchical temporal sequence modeling using bidirectional long short-term memory (BiLSTM) networks with attention pooling, and (4) classification with data augmentation and class-weighted loss functions to address class imbalance. Each component is designed to address specific challenges in automatic sleep staging and is described in detail in the following subsections.

**Fig 1:**
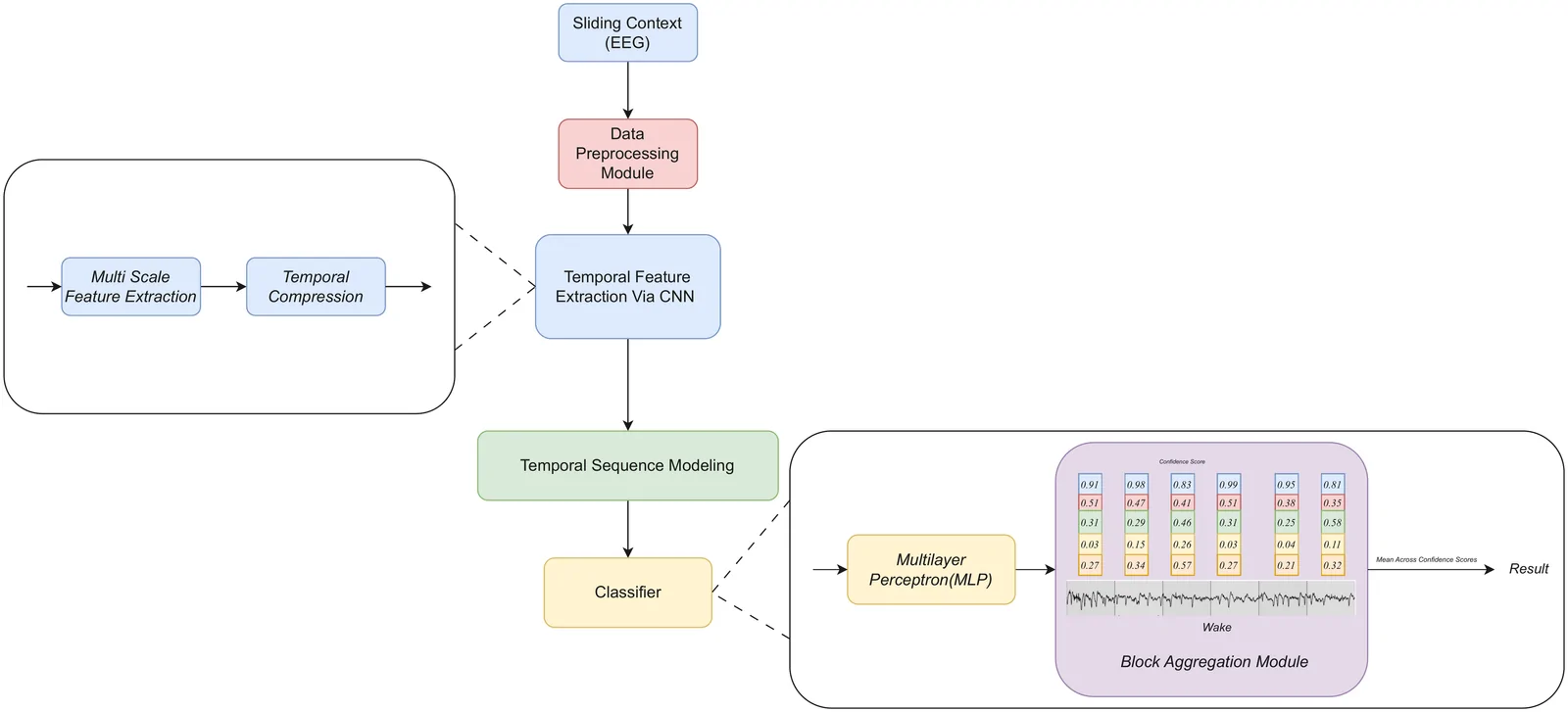
Overall overview of the TempoSleep pipeline. The end-to-end architecture includes EEG preprocessing with sliding context windows, multi-scale temporal feature extraction and compression via CNNs, hierarchical temporal sequence modeling, and final classification with block-level aggregation of confidence scores.

#### Data preprocessing.

We have employed some novel preprocessing methods in this area alongside previously used ones from earlier works [32]. As shown in Fig 2, trimmed signals were segmented into 30-second epochs. In addition, each of these epochs was divided into 6 non-overlapping 5-second sub-epochs to have high-resolution signals for subsequent analysis. Furthermore, the model has to be evaluated using 30-second epochs to be comparable with other papers, so we assigned each 5-second chunk a block identifier that is relevant to its parent. Thereby, in the classification module, predictions can be mapped back to the original epoch from which the sub-epochs originated. Instead of looking at each 5-second piece of data individually, a three-sub-epoch context window was applied to each central sub-epoch, and was grouped with the piece before it and the piece after it. This approach captures short-term temporal dependencies, and the contextual information surrounding the central segment can be used. For each three-sub-epoch window, the central sub-epoch’s label was assigned as the target.

**Fig 2:**
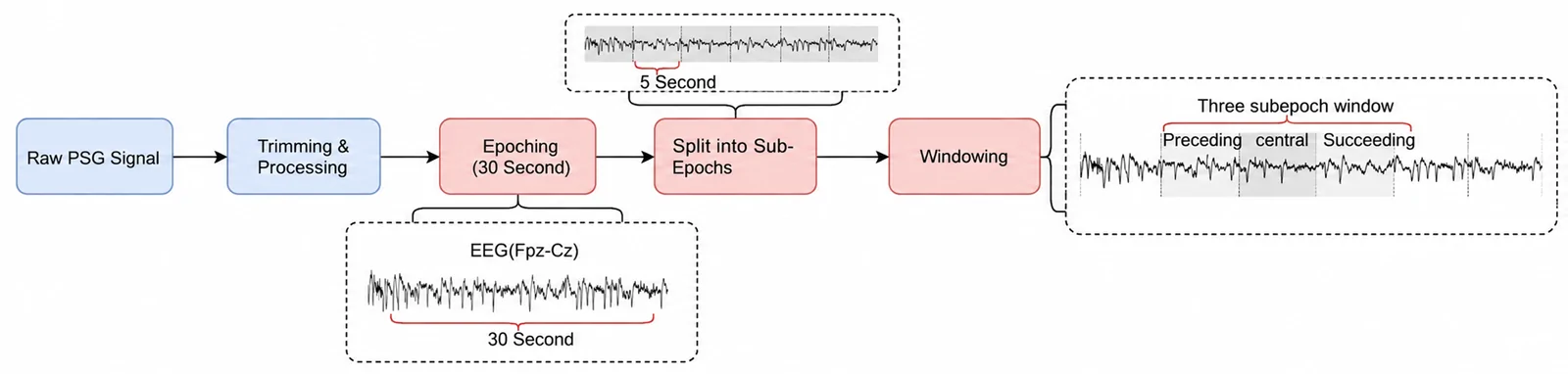
Structure of the preprocessing and sub-epoch windowing pipeline. The pipeline consists trimming of raw PSG recordings, segmentation into 30-second epochs, further division into non-overlapping 5-second sub-epochs, and construction of three-sub-epoch context windows (preceding, central, and succeeding) used for contextual modeling.

#### Temporal feature extraction via CNN.

The overall architecture of the temporal feature extraction module, including the multi-scale convolutional branches and temporal compression pathway, is illustrated in Fig 3. After the preprocessing stage, the data are passed to the *Temporal Feature Extraction via CNN* component. It is composed of two sub-blocks, namely *Multi-Scale Feature Extraction* and *Temporal Compression*.

**Fig 3:**
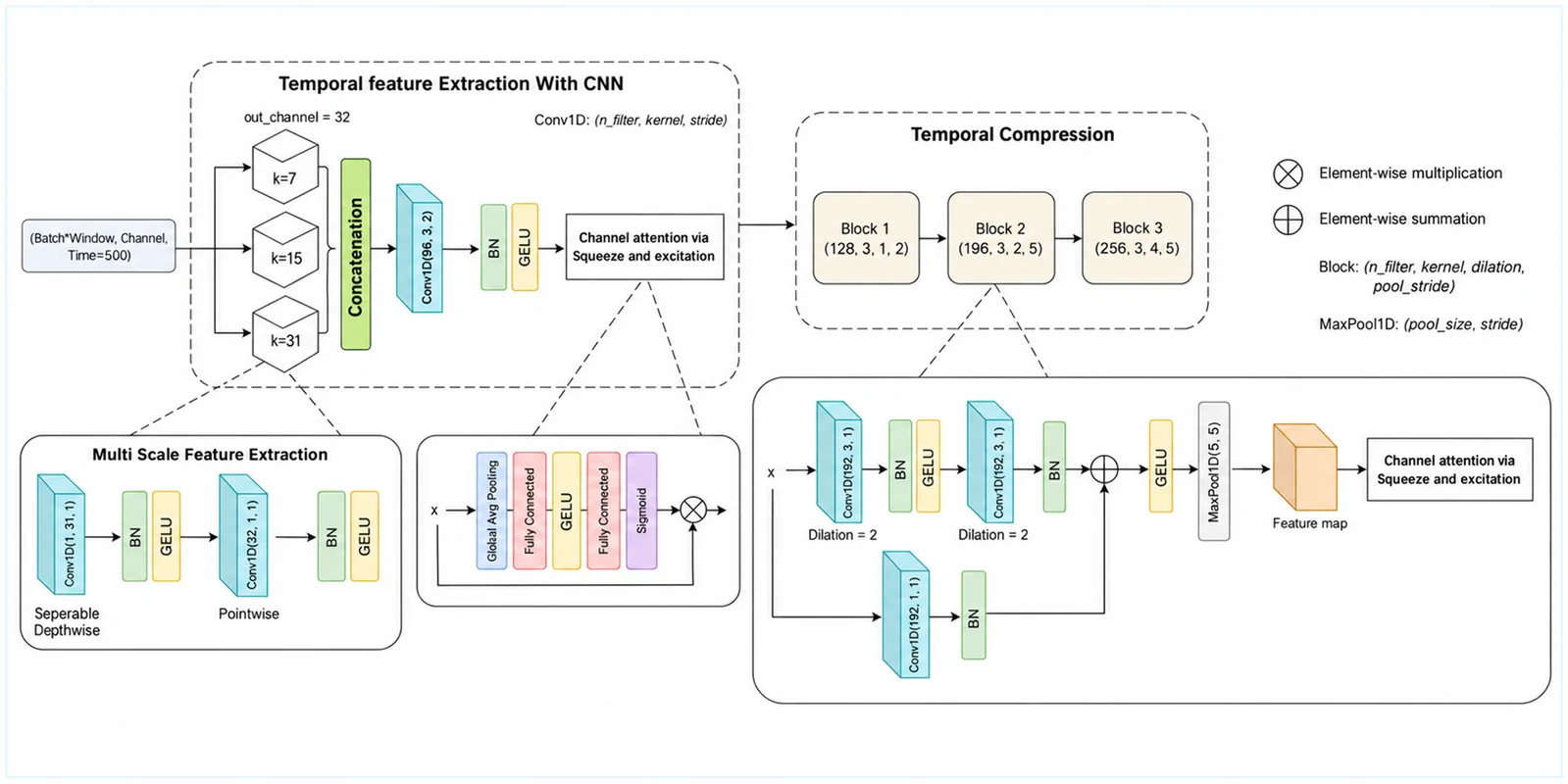
CNN-based temporal feature extraction and compression. The architecture includes multi-scale convolutional branches with different kernel sizes, depthwise separable convolutions, squeeze-and-excitation–based channel attention, and subsequent temporal compression blocks that reduce temporal resolution while expanding the feature dimension.

*All convolutions in this module are 1D, operating along the temporal dimension.*

The input and output tensors are defined as:


$$\begin{array}{c} 𝐗\in {\mathbb{R}}^{B\cdot W\times C_{in}\times T_{in}},\;𝐘\in {\mathbb{R}}^{B\cdot W\times C_{out}\times T_{out}}, \end{array}$$ (1)

where *B* is the batch size, *W* the number of windows, $C_{in}=1$ the number of input channels, and $T_{in}=500$ the number of samples per window. The output tensor has $C_{out}=256$ channels and $T_{out}=5$ latent time steps.

At the end of this component, the original 500-sample window (5 s at 100 Hz) is reduced to 5 latent time steps, corresponding to a temporal compression factor:


$$\begin{array}{c} T_{out}=\frac{T_{in}}{100}\;. \end{array}$$ (2)

Band-specific information (e.g., delta 0.5–4 Hz, theta 4–8 Hz, spindle 11–16 Hz) is first extracted by the convolutional branches prior to temporal downsampling; the latent sequence summarizes these learned features across time.

***Multi-Scale Feature Extraction*:** The first sub-block is the *Multi-Scale Feature Extraction* stage, designed to capture multi-scale temporal features across different frequency ranges. It employs three parallel convolutional branches with kernel sizes of 7, 15, and 31 samples, respectively, targeting short-, medium-, and long-term dependencies.

Each branch applies depthwise separable convolution [33] followed by a 1×1 pointwise convolution. The parameter counts are given by:


$$\begin{array}{c} {\text{Params}}_{\text{full}}=k\cdot C_{in}\cdot C_{out},\;{\text{Params}}_{\text{dw}}=k\cdot C_{in}+C_{in}\cdot C_{out}\;, \end{array}$$ (3)

where *k* denotes the kernel size. This design significantly reduces computation compared to full convolution. This operation also contributes to the lightweight nature of the proposed model. For a standard 1D convolution, the number of parameters is $kC_{in}C_{out}$, whereas for a depthwise separable convolution it is reduced to $kC_{in}+C_{in}C_{out}$. Therefore, the convolutional feature extractor reduces parameter growth while preserving multi-scale temporal representation. The outputs of the three branches are concatenated to form a unified feature map:


$$\begin{array}{c} 𝐅=\text{Concat}({𝐅}_{k=7},{𝐅}_{k=15},{𝐅}_{k=31})\;. \end{array}$$ (4)

Following concatenation, a Gaussian Error Linear Unit (GELU) [34] nonlinearity is applied, and a 3×1 convolution with stride 2 is used for early temporal pooling.

A squeeze-and-excitation (SE) [35] block is then employed to enhance channel-wise feature quality:


$$\begin{array}{c} 𝐬=\sigma \;\left(W_{2}\;\delta \;(W_{1}\cdot \text{GAP}(𝐔))\right),\;{𝐗}^{\prime }=𝐬⊙𝐔\;, \end{array}$$ (5)

where GAP denotes global average pooling, δ is the GELU activation, σ is the sigmoid function, and *r* = 8 is the reduction ratio.

The Multi-Scale Feature Extraction stage contains approximately $30.6\times 10^{3}$ parameters, most of which are concentrated in the reduction convolution stage. Overall, it achieves efficient multi-frequency receptive field fusion while maintaining a modest computational budget.

***Temporal Compression***: The second stage, *Temporal Compression*, is designed to reduce temporal resolution and expand feature channels, while capturing long-range dependencies with dilated convolutions [36]. Each of the three residual blocks contains two 1D convolutions, batch normalization, GELU activation, a residual (or projected) shortcut, max-pooling for downsampling, and a squeeze–and–excitation (SE) module for channel reweighting. The dilated 1D convolution used in this stage is defined as:


$$\begin{array}{c} y_{o}[t]=\sum_{i=1}^{C_{\text{in}}}\sum_{m=0}^{k-1}W_{o,i}[m]\;x_{i}[t-d\cdot m]\;. \end{array}$$ (6)

Each residual block applies two such convolutions, followed by normalization and activation. The residual unit with projection is given by:


$$\begin{array}{c} {𝐳}_{1}=\phi ({BN}_{1}({Conv}_{k_{1},d_{1}}(𝐱))), \end{array}$$ (7)

$$\begin{array}{c} {𝐳}_{2}={BN}_{2}({Conv}_{k_{2},d_{2}}({𝐳}_{1})), \end{array}$$ (8)

$$\begin{array}{c} 𝐲=\phi ({𝐳}_{2}+𝒫(𝐱)). \end{array}$$ (9)

After the residual operation, temporal downsampling is performed using max pooling:


$$\begin{array}{c} \tilde{𝐲}={MaxPool}_{s_{i}}(𝐲),\;T_{i}=\left\lfloor \frac{T_{i-1}}{s_{i}}\right\rfloor , \end{array}$$ (10)

We use three temporal strides, specifically $\left(s_{1},s_{2},s_{3})=(2,5,5\right)$. Applying these strides reduces the sequence length step by step: starting from *T*_0_ = 250, it becomes *T*_1_ = 125, then *T*_2_ = 25, and finally *T*_3_ = 5.

Across the convolutional blocks, the number of channels increases progressively. The model starts with *C*_0_ = 96 channels in the input stage, then expands to 128 in block 1, 192 in block 2, and reaches 256 channels in block 3.

Overall, the input sequence (which is 500 samples after preprocessing) is compressed down to just 5 latent steps. This corresponds to a temporal compression factor of 500 divided by 5, which equals 100. This temporal compression is important for computational efficiency because the recurrent module does not process the original 500-sample sequence directly. Instead, it operates on only 5 latent time steps. Since the computational cost of an LSTM is approximately *O*(*T H*(*D* + *H*)), where *T* is the sequence length, *D* is the input dimension, and *H* is the hidden size, reducing *T* before recurrent modeling substantially lowers the computational burden.

Because dilations (1,2,4) are used across blocks, the receptive field grows to cover a broader temporal context. The receptive field increment at block *i* is:


$$\begin{array}{c} \Delta {RF}_{i}=(k_{1}-1)\;d_{1}+(k_{2}-1)\;d_{2}, \end{array}$$ (11)

and accumulates as:


$$\begin{array}{c} {RF}_{i}={RF}_{i-1}+[(k_{1}-1)\;d_{1}+(k_{2}-1)\;d_{2}]\cdot \prod_{j=0}^{i-1}s_{j}, \end{array}$$ (12)

$$\begin{array}{c} s_{0}=2\;\;(\text{multi-scale stride}). \end{array}$$ (13)

Finally, this stage outputs:


$$\begin{array}{c} {𝐘}_{\text{res}}\in {\mathbb{R}}^{B\cdot W\times 256\times 5}, \end{array}$$ (14)

which is passed to the subsequent LSTM head for higher-level temporal modeling.

#### Temporal sequence modeling.

Fig 4 illustrates the hierarchical temporal sequence modeling block, which integrates an intra-window BiLSTM, an inter-window BiLSTM [37], and an additive attention pooling mechanism [38]. This block operates after the CNN feature extractor and is responsible for modeling both short- and long-range temporal dependencies.

**Fig 4:**
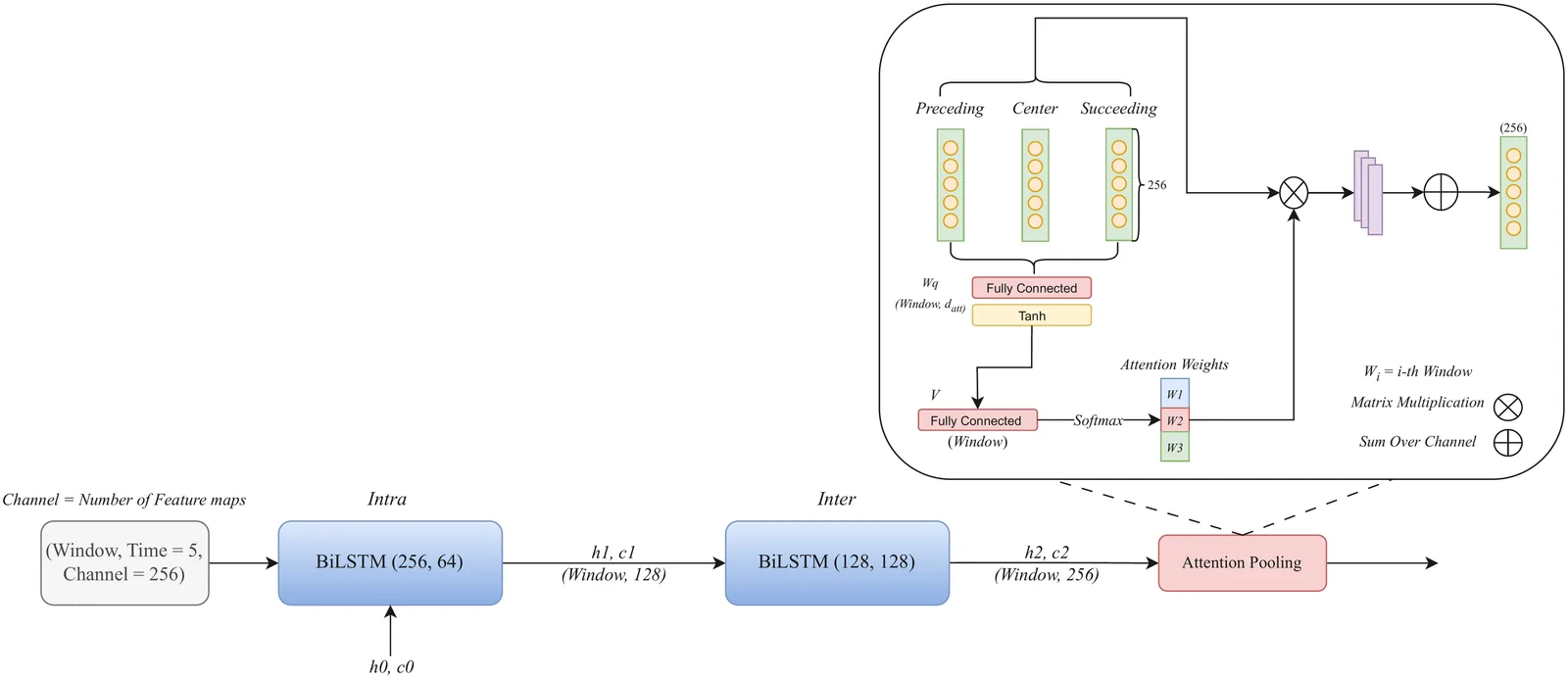
Hierarchical BiLSTM-based temporal sequence modeling. The intra-window BiLSTM for modeling short-term temporal dynamics, the inter-window BiLSTM for capturing contextual dependencies across neighboring sub-epochs, and the additive attention pooling mechanism used to aggregate window-level representations.

The input to this component (output of the previous module) is


$$\begin{array}{c} 𝐙\in {\mathbb{R}}^{B\cdot W\times F\times 5}, \end{array}$$ (15)

where *F* = 256 and the final dimension corresponds to the 5 latent timesteps per window. Here, *B* denotes the batch size and *W* denotes the number of context windows (set to *W* = 3 in our experiments). The temporal modeling stage yields a fixed-size embedding $𝐬\in {\mathbb{R}}^{2H_{2}}$ (here 2*H*_2_=256), which is used as input to the MLP classifier.

***Intra-window BiLSTM***: This module operates within each 5-step window. First, the input is permuted to match the format expected by PyTorch LSTMs:


$$\begin{array}{c} 𝐙\;\to \;{\mathbb{R}}^{B\cdot W\times 5\times F}. \end{array}$$ (16)

A bidirectional LSTM with hidden size *H*_1_ = 64 per direction is applied across the 5 latent timesteps. We take the last timestep output to obtain a per-window representation ${\tilde{𝐡}}_{i}\in {\mathbb{R}}^{2H_{1}}$. These representations capture fine-grained micro-temporal dynamics within each 5-step latent sequence, preserving short-range dependencies learned during compression.

***Inter-window BiLSTM***: The per-window representations are stacked back into a sequence ordered as past, center, and future windows:


$$\begin{array}{c} \tilde{𝐇}\in {\mathbb{R}}^{B\times W\times 2H_{1}}. \end{array}$$ (17)

This sequence forms the input to the inter-window BiLSTM. A second bidirectional LSTM is applied across the *W* windows with hidden size *H*_2_ = 128 per direction, producing


$$\begin{array}{c} 𝐔\in {\mathbb{R}}^{B\times W\times 2H_{2}}. \end{array}$$ (18)

This module models relationships between neighboring sub-epochs (sleep micro-architecture).

***Additive Attention Pooling***: Given 𝐔, we pool information across the *W* windows into a single vector. For each window *t*,


$$\begin{array}{c} {𝐬}_{t}=\tanh\;(W_{q}{𝐔}_{t}),\;e_{t}={𝐯}^{⊤}{𝐬}_{t}, \end{array}$$ (19)

where $W_{q}\in {\mathbb{R}}^{2H_{2}\times d_{\text{att}}}$ and $𝐯\in {\mathbb{R}}^{d_{\text{att}}}$. Attention weights are obtained via a softmax across windows:


$$\begin{array}{c} \alpha_{t}=\frac{\exp(e_{t})}{\sum_{\tau =1}^{W}\exp(e_{\tau })}. \end{array}$$ (20)

The sequence is then aggregated by a weighted sum, yielding the fixed-length embedding


$$\begin{array}{c} 𝐬=\sum_{t=1}^{W}\alpha_{t}\;{𝐔}_{t}\;\in \;{\mathbb{R}}^{2H_{2}}. \end{array}$$ (21)

With *d*_att_ = 64, the attention weights have shape $\alpha \in {\mathbb{R}}^{B\times W}$, and the pooled vector has shape $𝐬\in {\mathbb{R}}^{2H_{2}}$. Each weight in α indicates the contribution of a sub-epoch to the final inference and is retained for analysis. The attention parameters $\left(W_{q},𝐯\right)$ are learned jointly with the rest of the network; no auxiliary supervision is used. Finally, 𝐬 is passed to the MLP classifier.

#### MLP classifier.

After the LSTM layer, the features extracted by that layer are passed to a final classification module. This module is primarily employed to transform the high-level temporal features into the final sleep stage probabilities. The Hierarchical LSTM head produces a final feature vector of 256 dimensions (2 × 128 for the bidirectional hidden states) for each input sequence. Before this module, a dropout and normalization layer is used to prevent overfitting and to stabilize the learning process by normalizing the features across the entire feature dimension for each sample. These normalized features are passed to an MLP for the final classification.

The proposed MLP consists of two hidden layers and a final output layer. The first hidden layer is a linear layer that maps the 256-dimensional input to 512 neurons, followed by batch normalization, GELU, and dropout. In the second hidden layer, we employ a linear layer that maps 512 to 256 neurons, again followed by batch normalization, GELU, and dropout.

In the final output layer, a linear layer maps the 256-dimensional input to 5 neurons. Each of these neurons represents one of the Wake, N1, N2, N3, or REM classes. The output of this MLP is a vector of logits for each sleep stage, from which the final sleep stage prediction is derived.

#### Loss function.

We employed weighted cross-entropy for this multi-class classification problem. We used this approach because it is a standard loss function for multi-class classification, as it measures the dissimilarity between predicted probability distributions and true labels.

The main issue with sleep datasets is that they suffer from severe class imbalance. To address this, we employed weighted cross-entropy, where each class is weighted inversely proportional to its frequency in the training set. Additionally, we applied weight decay (L2 regularization) to the model parameters in order to reduce overfitting and improve generalization.

The weighted loss with weight decay is computed as:


$$\begin{array}{c} ℒ=-\sum_{b=1}^{B}\sum_{k=1}^{K}w_{k}\;y_{b,k}\;\log{\hat{p}}_{b,k}\;+\;\lambda \sum_{j}{\left\|\theta_{j}\right\|}_{2}^{2}, \end{array}$$ (22)

where $w_{k}$ is the weight for class *k*, $y_{b,k}$ is the ground-truth one-hot label for sample *b*, ${\hat{p}}_{b,k}$ is the predicted probability for class *k*, $\theta_{j}$ represents the trainable model parameters, and λ is the weight decay coefficient.

## Results

To evaluate TempoSleep, we performed a series of experiments to analyze it from several aspects. First, we examined our overall performance under a cross-validation protocol and analyze the outcomes across different stages. Secondly, we compared the overall and per-class performance of our method with established baselines using standard evaluation metrics to show its distinctive advantages. Finally, we performed an ablation study to show the contribution of individual components of our method to the overall performance.

### Experimental setups

To evaluate our proposed model, we performed 5-fold cross-validation using Stratified Group K-Fold cross-validation to ensure subject independence and prevent data leakage by grouping epochs based on block identifiers according to our preprocessing method. The model was trained using the Adam optimizer along with a learning rate scheduler and weight decay for regularization. Early stopping was applied after 30 epochs without improvement in validation accuracy. As mentioned previously, Due to the imbalanced dataset, the model was trained with weighted cross-entropy loss due to the imbalanced dataset. All experiments were performed on an NVIDIA A100 GPU via the Google Colab Pro Service. The hyperparameters are detailed in Table 2.

**Table 2:** Experimental hyperparameters.

Hyperparameter	Value
Learning rate (lr)	$1\times 10^{-4}$
Weight decay	$1\times 10^{-4}$
Batch size	128
Maximum epochs	100
Early stopping patience	30
Learning rate scheduler	ReduceLROnPlateau
Scheduler decay factor	0.5
Scheduler patience	7 epochs

### Evaluation metrics

Overall performance of our model was evaluated using metrics commonly employed in previous studies, including accuracy (Acc.), macro-average F1-score (MF1), Cohen’s kappa coefficient (K), sensitivity (Sens.), and specificity (Spec.). Additionally, for per-class analysis, we used per-class F1-score (*F*_1_), per-class precision (*P*_rec_), per-class sensitivity (*P*_sens_), and per-class area under the receiver operating characteristic curve (AUROC). We also presented detailed results for each class using the confusion matrix.

### Overall cross-validated performance

Overall performance of the 5-fold cross-validation was detailed in Tables 3–5.

**Table 3:** Per-class performance on SleepEDF-20 and SleepEDF-78 using the Fpz–Cz EEG channel.

Dataset	Class	F1	Precision	Sensitivity	Specificity	AUROC
SleepEDF-20	WAKE	94.80	95.70	93.90	99.00	99.70
	N1	61.70	68.90	56.00	98.20	95.90
	N2	91.40	90.80	91.90	93.30	98.00
	N3	89.70	89.70	89.70	98.40	99.40
	REM	89.80	87.30	92.40	97.00	99.10
SleepEDF-78	WAKE	95.00	96.00	94.00	98.60	99.46
	N1	59.00	64.00	54.00	97.00	93.29
	N2	87.00	86.00	89.00	94.00	97.06
	N3	83.00	83.00	84.00	98.00	99.28
	REM	85.00	85.00	88.00	96.00	98.72

**Table 4:** Per-class comparison of F1, Precision, and Sensitivity on SleepEDF-20 and SleepEDF-78 using the Fpz–Cz EEG channel.

Model	Stage	SleepEDF-20			SleepEDF-78		
		F1	Precision	Sensitivity	F1	Precision	Sensitivity
TempoSleep (Proposed method)	WAKE	**94.8**	**95.7**	**93.9**	**95.0**	**96.0**	**94.0**
	N1	**61.7**	**68.9**	**56.0**	**59.0**	**64.0**	**54.0**
	N2	**91.4**	90.8	**91.9**	**87.0**	86.0	**89.0**
	N3	89.7	89.7	89.7	83.0	83.0	84.0
	REM	**89.8**	87.3	**92.4**	**85.0**	85.0	**88.0**
AttnSleep [25]	WAKE	89.7	89.6	89.7	92.0	92.3	91.8
	N1	42.8	47.1	39.1	42.1	45.3	39.2
	N2	88.8	89.1	88.6	85.0	83.5	86.5
	N3	**90.2**	**90.7**	89.8	**82.1**	**82.3**	82.0
	REM	79.0	76.1	82.2	74.2	73.1	75.3
SleepEEGNet [28]	WAKE	89.1	87.8	90.5	91.7	–	–
	N1	52.1	50.0	54.5	44.0	–	–
	N2	86.7	**91.2**	82.7	82.4	–	–
	N3	85.1	81.6	88.8	73.6	–	–
	REM	85.0	81.6	88.7	76.0	–	–
U-Time [39]	WAKE	87.0	–	–	92.0	–	–
	N1	52.0	–	–	51.0	–	–
	N2	86.0	–	–	84.0	–	–
	N3	84.0	–	–	75.0	–	–
	REM	84.0	–	–	80.0	–	–
DeepSleepNet [26]	WAKE	84.7	86.0	83.4	–	–	–
	N1	46.6	43.5	50.1	–	–	–
	N2	85.9	90.5	81.7	–	–	–
	N3	84.8	77.1	**94.2**	–	–	–
	REM	82.4	80.9	83.9	–	–	–
SleepEGAN [29]	WAKE	91.7	–	–	93.1	–	–
	N1	53.6	–	–	51.7	–	–
	N2	89.2	–	–	85.8	–	–
	N3	89.1	–	–	81.2	–	–
	REM	86.1	–	–	82.0	–	–
SleepFocalNet [40]	WAKE	93.4	94.0	92.8	93.7	92.9	94.6
	N1	55.4	61.4	50.5	52.3	60.4	46.2
	N2	90.4	89.1	91.7	86.9	84.9	89.1
	N3	89.5	89.4	89.6	80.1	84.2	76.5
	REM	89.1	88.8	89.5	87.1	84.5	89.9

**Table 5:** Overall performance comparison on SleepEDF-20 and SleepEDF-78 using the Fpz–Cz EEG channel. Best and second-best results are highlighted in bold and underlined, respectively.

Model	Acc.	Kappa	Macro F1	Sens.	Spec.	Acc.	Kappa	Macro F1	Sens.	Spec.
	SleepEDF-20					SleepEDF-78				
TempoSleep (Proposed method)	**89.72**	**85.85**	**85.46**	**84.78**	**97.16**	**86.41**	81.25	**81.97**	**81.86**	**96.39**
SeqSleepNet [27]	82.20	79.80	78.40	78.00	96.10	82.60	76.00	76.40	76.30	95.40
AttnSleep [25]	84.40	79.00	78.10	–	–	81.30	74.30	75.10	–	–
SleepEEGNet [28]	84.26	79.00	79.66	–	–	80.03	73.00	73.55	–	–
U-Time [39]	82.10	78.00	78.80	–	–	82.10	73.00	76.50	–	–
DeepSleepNet [26]	80.80	73.10	74.20	–	–	78.50	70.20	75.30	75.00	95.10
XSleepNet [42]	86.30	81.30	80.60	80.20	96.40	84.00	77.80	77.90	77.50	95.70
SleepGCN [41]	86.96	81.68	81.81	82.85	96.54	83.79	77.62	76.19	75.53	95.67
SleepEGAN [29]	86.80	82.00	81.90	–	–	83.80	**82.00**	78.70	–	–
SleepFocalNet [40]	88.50	84.10	83.60	–	–	85.50	80.00	79.80	–	–

The corresponding learning curves, illustrating the evolution of training and validation accuracy across epochs, are shown in Fig 5. The results indicate that the proposed model achieves stable performance with low variance across folds.

**Fig 5:**
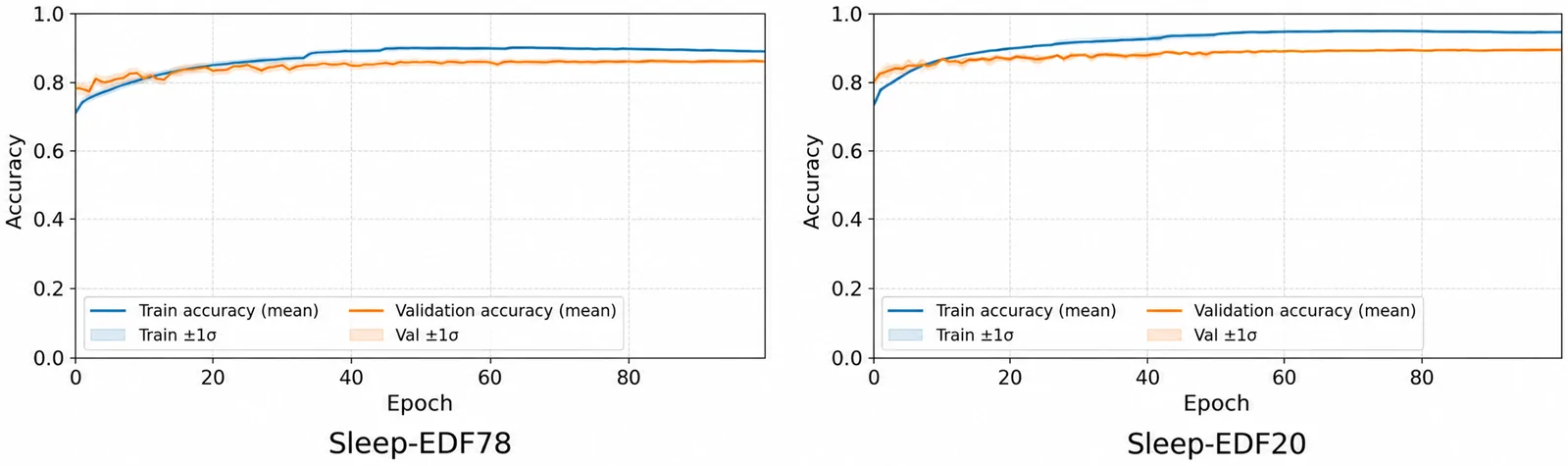
Cross-validation learning curves showing training and validation accuracy on SleepEDF-20 and SleepEDF-78. The mean training and validation accuracy across epochs obtained from 5-fold cross-validation, with shaded regions indicating ±1 standard deviation, illustrating the convergence behavior and stability of the proposed model.

The normalized confusion matrices in Fig 6 show consistently high classification performance on both SleepEDF-20 and SleepEDF-78, particularly for Wake, N2, N3, and REM. N1 remains the most challenging stage, with most misclassifications occurring with N2 and Wake. N3 is primarily confused with N2, while REM and Wake show relatively limited confusion with other stages.

**Fig 6:**
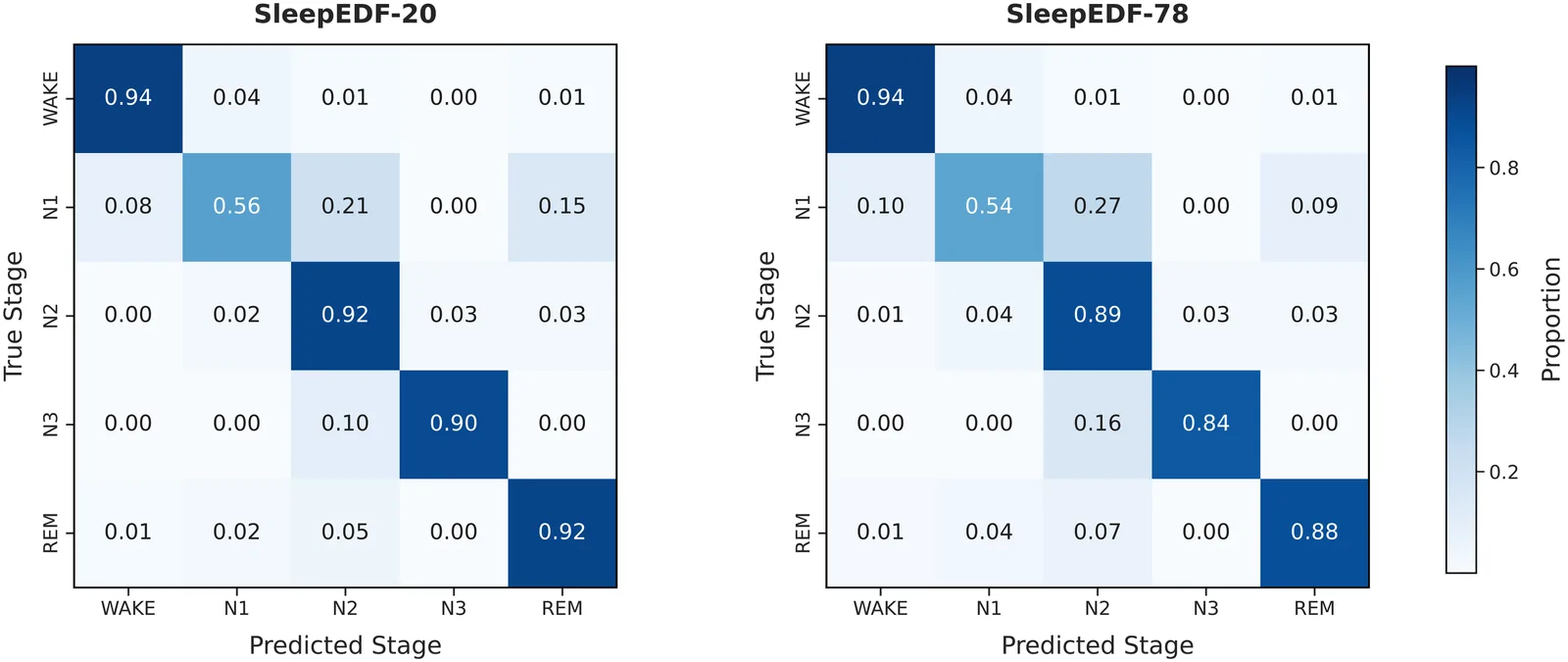
Normalized confusion matrices of the proposed model on SleepEDF-20 and SleepEDF-78.

A comparison between expert-annotated and model-predicted hypnograms is shown in Fig 7.

**Fig 7:**
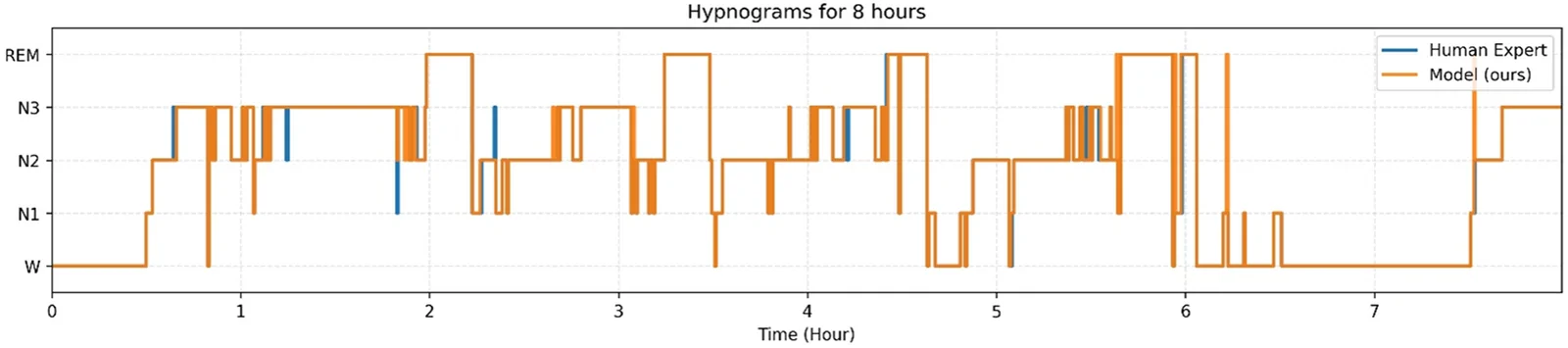
Hypnograms over 8 hours comparing human expert annotations to our model predictions. The comparison of sleep stage sequences annotated by a human expert with those predicted by the proposed model across a complete overnight recording from a single subject in the SleepEDF-20 dataset.

Our method is compared with the following baselines:

1. DeepSleepNet used CNN layers to extract time-invariant features from raw single-channel EEG, followed by BiLSTM layers to model temporal dependencies between epochs. The model was trained in two stages (pretraining and fine-tuning) to capture both short- and long-term sleep patterns [26].
2. SeqSleepNet reformulated the sleep staging task as a sequence-to-sequence classification problem. It used parallel filterbanks and BiRNN layers to extract time-frequency features [27].
3. AttnSleep used multi-scale CNN branches with attention to capture multi-scale EEG features while emphasizing salient patterns [25].
4. U-Time used a fully convolutional U-Net followed by an LSTM layer to capture temporal dependencies [39].
5. SleepEEGNet combined CNNs and BiRNNs to extract time-frequency domain features and capture temporal dependencies, respectively [28].
6. SleepEGAN introduced a generative adversarial network (GAN) framework for sleep staging to augment minority sleep stages, such as N1 [29].
7. SleepGCN introduced a graph convolutional framework to explicitly model sleep transition rules, combining a residual network with an LSTM branch for feature extraction [41].
8. SleepFocalNet used focal modulation with multi-scale feature extraction via CNNs [40].
9. XSleepNet leveraged BiRNNs to fuse raw signal data with time–frequency image features, creating a joint representation to support sleep stage prediction [42].

To evaluate the computational efficiency of TempoSleep, we measured its model complexity and inference time. As summarized in Table 6, the proposed model contains 1.515 million trainable parameters with a model size of 5.802 MB and requires approximately 1.514 GMACs (3.028 GFLOPs) per inference. The average inference time was 4.01±1.95 ms per 30-second epoch.

**Table 6:** Computational profile of the proposed model.

Metric	Value
Trainable parameters	1.515 M
Model size	5.802 MB
Computational complexity	3.028 GFLOPs / 1.514 GMACs
Inference time per 30-s epoch	4.01±1.95 ms
Training time (5-fold CV)	5 h 30 min

Training and inference times were measured on an NVIDIA A100 GPU.

As summarized in Table 5, TempoSleep outperforms all baselines in terms of every metric on the SleepEDF-20 dataset, achieving the highest accuracy (89.72%), kappa (85.85), macro-F1 (85.46%), sensitivity (84.78%), and specificity (97.16%). A similar performance trend is observed on the SleepEDF-78 dataset, as reported in Table 5. As shown in the per-class analysis in Table 4, our method achieves the highest F1-score and sensitivity in most sleep stages, with only limited exceptions where AttnSleep, DeepSleepNet, SleepEEGNet, or SleepFocalNet perform marginally better in a single metric. Remarkably, our method demonstrates significant improvement in the challenging N1 stage, achieving F1-scores of 61.7% and 59.0% on the SleepEDF-20 and SleepEDF-78 datasets, respectively. Overall, these results demonstrate that our proposed method consistently outperforms existing single-channel EEG methods in both global and class-level performance.

### Ablation study

This section presents the ablation study applied to examine the contribution of each proposed architecture using the SleepEDF-20 dataset. As illustrated in Table 7, a clear view of each component’s contribution is provided, including multi-scale convolution blocks, temporal compression, BiLSTM-based sequence modeling, and ultimately data augmentation. As a baseline model, the multi-scale feature extraction module was examined and achieved 84.39% accuracy and 80.60% macro-F1 score. These results show that using multi-scale kernels improves performance by extracting features at different temporal resolutions. Adding the temporal compression module significantly increased accuracy to 86.13%. Temporal downsampling represented each signal with compact feature map that retains rich information while expanding model’s receptive field. Further enhancement is achieved with the integration of the hierarchical temporal modeling block, which consists of intra-window BiLSTM, inter-window BiLSTM, and additive attention mechanisms. This resulted in 88.53% accuracy with 83.60% macro-F1 score. The results highlight that capturing contextual relationships is particularly important for distinguishing transitional and ambiguous sleep stages, such as N1 and REM. Finally, by adding data augmentation techniques, the complete model reached the highest performance level with 89.72% accuracy and a macro-F1 score of 85.46%. The provided data augmentation techniques significantly enhanced the model’s ability to generalize, particularly for the N1 stage, which resulted in a 2.7% increase in the N1 class-wise F1 score. While temporal compression and hierarchical sequence modeling accounted for the most substantial improvements, data augmentation added the robustness necessary to achieve the best performance across all evaluated metrics.

**Table 7:** Ablation study on SleepEDF-20.

Methods	Accuracy	Macro F1	Kappa	Sensitivity	Specificity	N1-class F1
Multi-Scale Feature Extraction Only	84.39	80.60	79.16	84.03	96.21	53.50
Multi-Scale Feature Extraction + Temporal Compression	86.13	79.85	80.30	76.37	95.68	57.00
Multi-Scale Feature Extraction + Temporal Compression + Temporal Sequence Modeling	88.53	83.60	84.08	80.90	96.10	59.00
Multi-Scale Feature Extraction + Temporal Compression + Temporal Sequence Modeling + Augmentation	**89.72**	**85.46**	**85.85**	**84.78**	**97.16**	**61.70**

To assess whether the incremental accuracy improvements were statistically meaningful, paired t-tests were performed on the fold-wise results of successive model configurations. As shown in Table 8, all incremental improvements were statistically significant at the 0.05 significance level (*p* < 0.05), with large effect sizes according to Cohen’s $d_{z}$. These results provide statistical support for the contribution of temporal compression, temporal sequence modeling, and data augmentation to the final model performance.

**Table 8:** Paired t-test results for the incremental ablation comparisons at a significance level of 0.05.

Added Component	Before (Mean ± SD)	After (Mean ± SD)	Mean Diff.	*p*-value	Decision (*H*_0_)	Cohen’s $d_{z}$
Temporal Compression	84.39 ± 0.22	86.13 ± 0.35	+1.74	0.0010	Rejected	3.20
Temporal Sequence Modeling	86.13 ± 0.35	88.53 ± 0.32	+2.40	<0.001	Rejected	6.96
Data Augmentation	88.53 ± 0.32	89.72 ± 0.29	+1.19	<0.001	Rejected	3.40

Overall, the ablation study demonstrates that each component makes a meaningful contribution to the proposed model’s final performance.

## Discussion

The findings demonstrate that the proposed method effectively addresses several key challenges in automatic single-channel EEG sleep staging, particularly the need to capture temporal information at multiple scales and the persistent class-imbalance problem associated with the N1 stage. Across the evaluated benchmarks, the proposed framework achieves strong overall and class-level performance while relying only on the Fpz–Cz EEG channel. The context-aware preprocessing strategy divides conventional 30-second epochs into finer sub-epoch segments and incorporates neighboring information, allowing the model to represent short-term transitions together with their surrounding temporal context. This design is complemented by multi-scale feature extraction, temporal compression, and hierarchical sequence modeling, which together provide a structured mechanism for capturing local EEG characteristics and longer-range dependencies.

The contribution of the major architectural components is supported by the ablation results in Table 7. Starting from the multi-scale feature extraction module, which achieved an accuracy of 84.39%, the addition of temporal compression increased accuracy to 86.13%. Incorporating hierarchical temporal sequence modeling further improved accuracy to 88.53%, while the inclusion of data augmentation produced the complete model performance of 89.72% accuracy and 85.46% macro-F1. The N1 F1-score also increased progressively from 53.5% with multi-scale feature extraction alone to 61.7% in the complete configuration. Moreover, the paired statistical comparisons in Table 8 showed that the incremental accuracy improvements associated with temporal compression, temporal sequence modeling, and data augmentation were statistically significant. These results indicate that the final performance does not arise from a single component but from the complementary contributions of compact multi-scale representation learning, temporal compression, hierarchical contextual modeling, and augmentation.

The stability of the proposed framework is further supported by Fig 5, where the training and validation curves converge steadily with relatively low variance across folds. This behavior indicates consistent learning and suggests that the model generalizes well under the subject-independent cross-validation protocol. As shown in the confusion matrices (Fig 6) and the per-class results (Table 3), the model performs reliably in predicting the Wake, N2, N3, and REM stages. In addition, its performance on the N1 stage surpasses that of the compared single-channel EEG baselines. Except for N1, the remaining stages generally exhibit more distinctive and stable EEG characteristics, which facilitates higher sensitivity and specificity.

The confusion matrices further reveal that most misclassifications occur between physiologically adjacent sleep stages rather than between clearly distinct sleep states. The confusion between N2 and N3 is consistent with the progressive increase in slow-wave activity during non-REM sleep, whereas the occasional confusion between Wake and N1 and between Wake and REM may be explained by similarities in their low-amplitude, mixed-frequency EEG characteristics and by the gradual physiological transitions between neighboring sleep stages [43]. These observations indicate that the residual errors are not randomly distributed across the sleep-stage space but are concentrated primarily at physiologically plausible boundaries between neighboring states.

Nevertheless, N1 remains the most challenging stage to classify, consistent with previous studies. This difficulty can be attributed to its inherently transitional nature and to the substantial overlap between N1 and neighboring stages, particularly wakefulness and N2. To further examine the patterns associated with N1 misclassifications, Table 9 compares the spectral characteristics of correctly classified and misclassified N1 epochs. N1 epochs misclassified as N3 showed substantially higher relative delta power and lower spectral entropy than correctly classified N1 epochs, while those misclassified as N2 also showed increased relative delta power. N1 epochs misclassified as REM exhibited relatively higher theta power, whereas those misclassified as Wake showed a spectral profile closer to correctly classified N1 epochs. Overall, these findings indicate distinct spectral patterns across N1 classification outcomes, consistent with the transitional and less distinctive nature of N1.

**Table 9:** Spectral characteristics of correctly classified and misclassified N1 epochs. Values are reported as mean ± standard deviation. “/” separates the true label from the model prediction.

N1 Outcome	Relative Delta Power	Relative Theta Power	Relative Alpha Power	Relative Sigma Power	Relative Beta Power	Spectral Entropy
N1 / N1	0.6659 ± 0.1395	0.1442 ± 0.0750	0.0745 ± 0.0519	0.0349 ± 0.0197	0.0735 ± 0.0590	0.6762 ± 0.1101
N1 / Wake	0.6800 ± 0.1696	0.1230 ± 0.0910	0.0867 ± 0.0736	0.0330 ± 0.0215	0.0723 ± 0.0591	0.6276 ± 0.1478
N1 / N2	0.7559 ± 0.1121	0.1178 ± 0.0586	0.0474 ± 0.0306	0.0357 ± 0.0293	0.0362 ± 0.0458	0.6401 ± 0.0913
N1 / N3	0.9082 ± 0.0403	0.0436 ± 0.0103	0.0162 ± 0.0065	0.0088 ± 0.0065	0.0202 ± 0.0191	0.4836 ± 0.0430
N1 / REM	0.6557 ± 0.1177	0.1666 ± 0.0661	0.0740 ± 0.0474	0.0336 ± 0.0182	0.0580 ± 0.0435	0.6980 ± 0.1040

Another important consideration when interpreting N1 performance is the inherent variability of N1 annotations. Previous studies have reported poor inter-rater agreement among sleep-scoring experts for this stage, which may introduce additional uncertainty into supervised learning frameworks. Since the SleepEDF-20 and SleepEDF-78 datasets are publicly available benchmark datasets widely used in automatic sleep staging research, we followed their original expert annotations and standard evaluation protocols. Nevertheless, potential annotation variability should be considered when interpreting N1 classification performance. Future studies using consensus-based annotations or larger clinical datasets with assessments from multiple experts may provide further insight into this challenge.

Despite these difficulties, the proposed model achieves an F1-score of 61.7% for N1 on SleepEDF-20 and 59.0% on SleepEDF-78 using only single-channel EEG. These results compare favorably with the N1 performance of the evaluated baselines and are particularly important because improvements in this minority and transitional stage are often difficult to obtain without sacrificing performance in the more prevalent classes. The ablation analysis further shows that N1 performance increases progressively as temporal compression, hierarchical sequence modeling, and augmentation are incorporated, supporting the value of combining contextual modeling with strategies designed to improve learning from minority-stage samples.

Beyond discrimination performance, understanding whether the model relies on physiologically meaningful EEG information is also important. Fig 8 shows a representative example illustrating how the model assigns importance to different EEG segments. The highlighted regions correspond to characteristic EEG patterns associated with the respective sleep stages, including alpha activity during wakefulness, theta activity during N1, spindles and K-complexes during N2, delta activity during N3, and mixed low-voltage activity with sawtooth waves during REM. The segment-importance visualization therefore provides a qualitative view of model behavior and suggests that the model attends to EEG regions containing physiologically relevant sleep-stage characteristics rather than relying exclusively on arbitrary signal segments. Although such visualization should not be interpreted as a complete mechanistic explanation of the network, its correspondence with characteristic sleep-related EEG patterns provides useful qualitative evidence about the regions influencing the model’s decisions.

**Fig 8:**
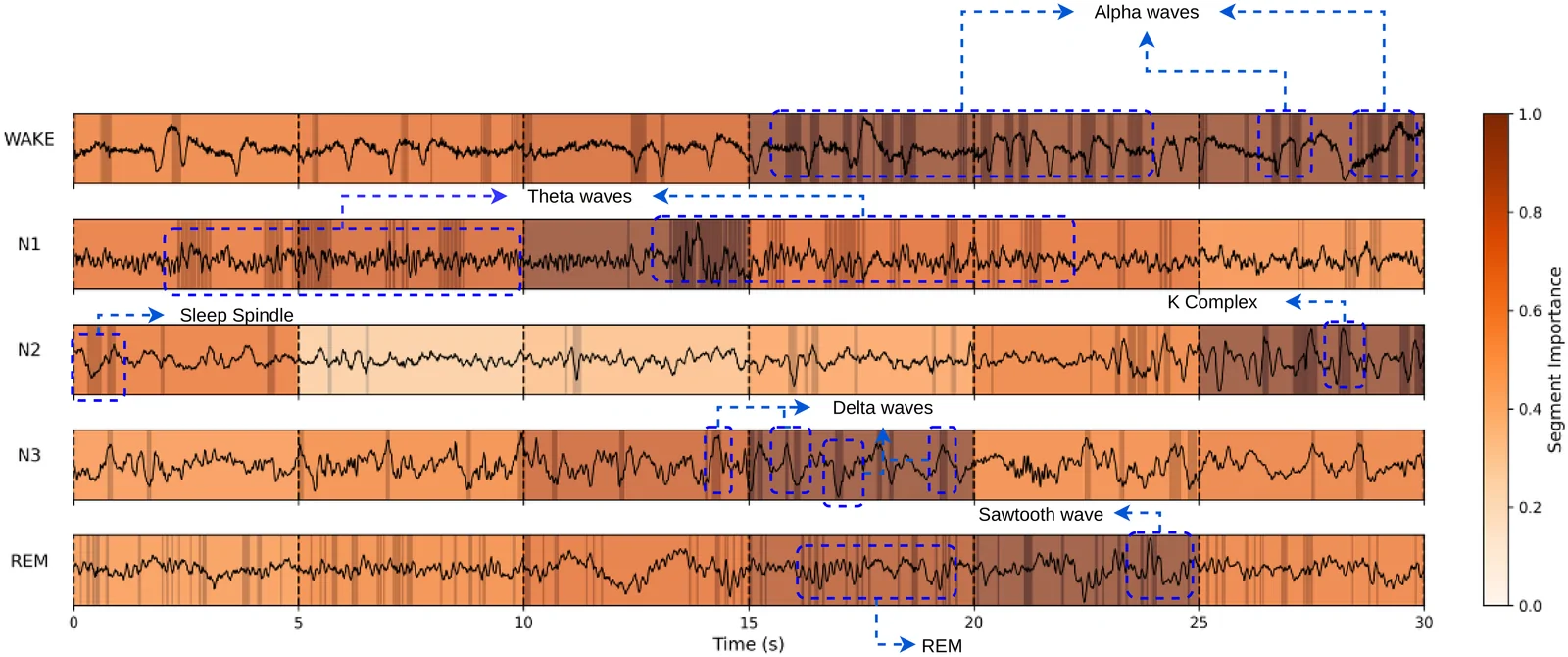
Segment-importance visualization for representative EEG segments across sleep stages from a single recording. The darker regions indicate higher contribution to the model’s decision, highlighting characteristic physiological patterns such as theta activity, sleep spindles, K-complexes, delta waves, and sawtooth waves.

Prediction reliability represents another important consideration, particularly for eventual clinical applications. Fig 9 shows that, even for the challenging N1 stage, the predicted confidence scores are relatively well calibrated.

**Fig 9:**
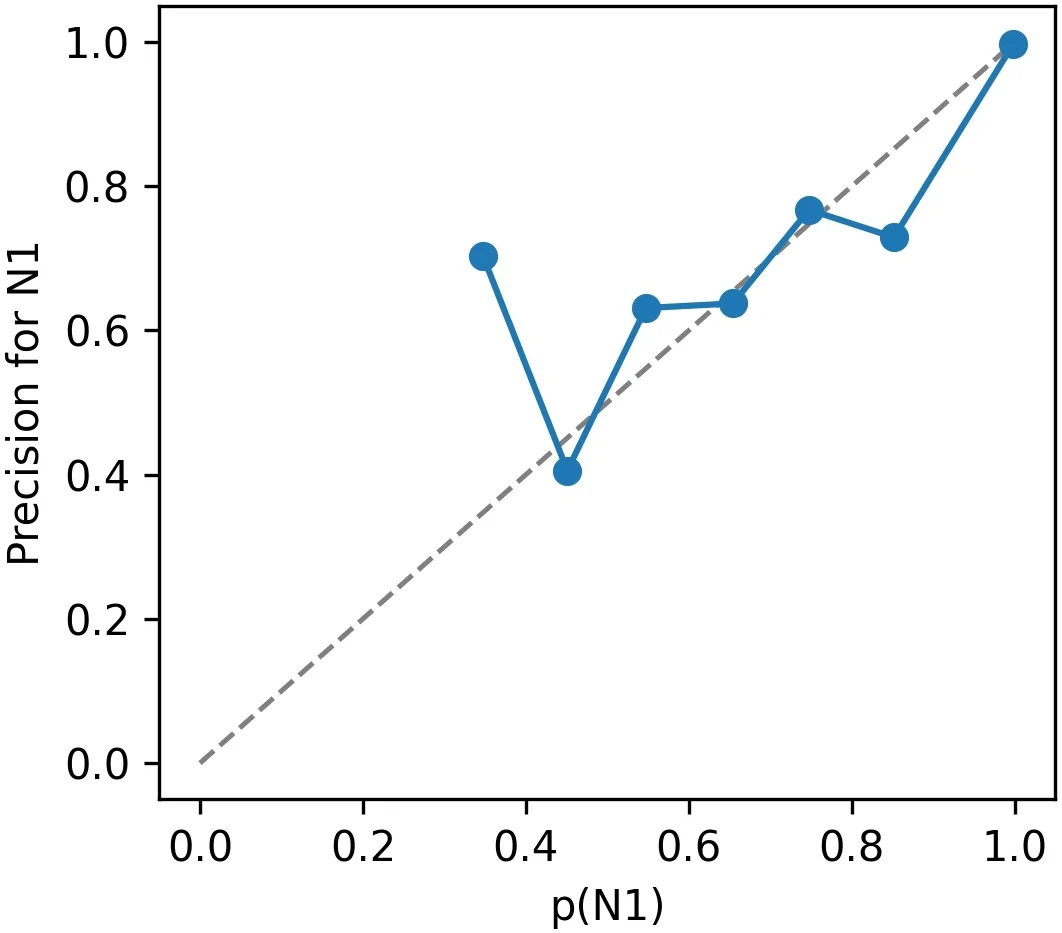
Reliability diagram for the N1 sleep stage. The calibration behavior of the model specifically for the N1 class.

The overall reliability diagram in Fig 10 further demonstrates good calibration across all sleep stages, with a low Expected Calibration Error of 0.010. Thus, the proposed framework provides not only strong discrimination performance but also confidence estimates that are generally consistent with the observed prediction outcomes.

**Fig 10:**
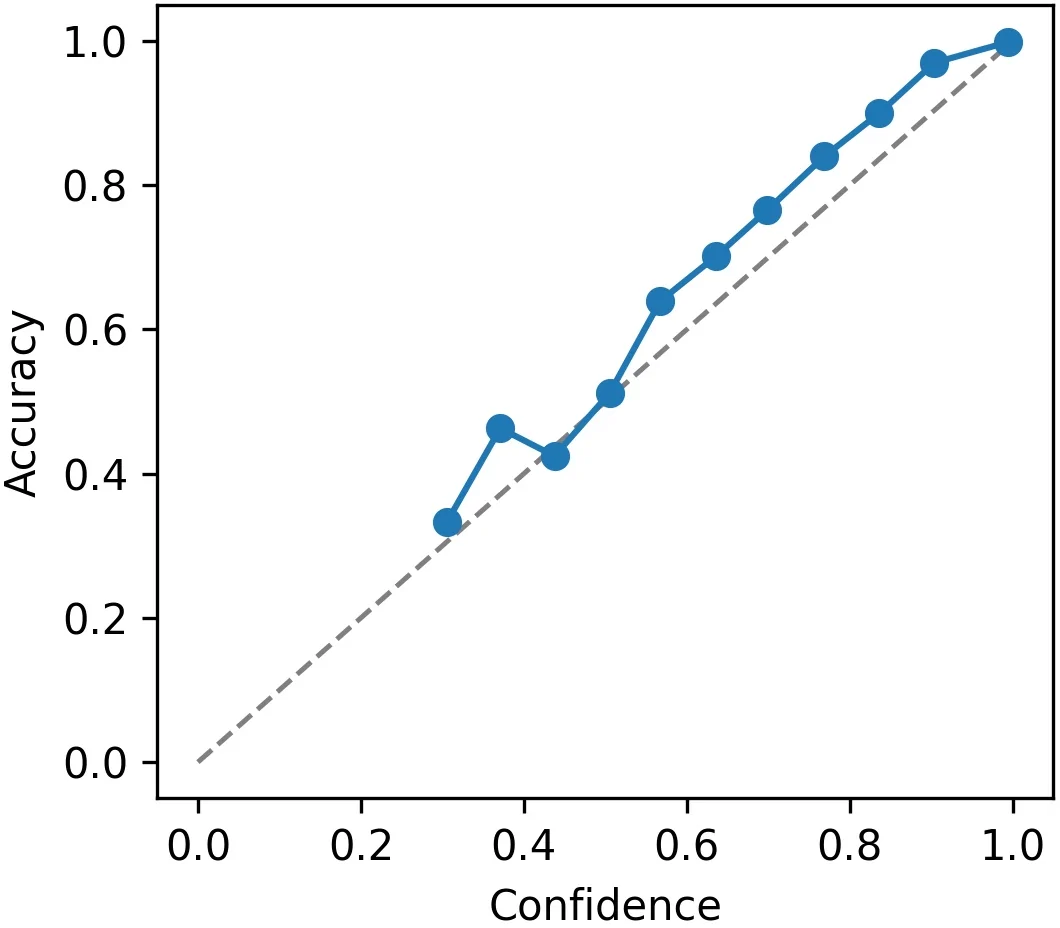
Reliability diagram for overall predictions. The relationship between predicted confidence and empirical accuracy across confidence bins, assessing the calibration quality of the proposed model over all sleep stages.

Together, the segment-importance visualization and calibration analysis complement the conventional performance metrics by providing additional information about both the qualitative behavior of the model and the reliability of its predictions.

The computational profile of the model also supports the objective of maintaining an efficient architecture. The proposed network contains approximately 1.515 million trainable parameters, occupies approximately 5.8 MB, and requires approximately 3.028 GFLOPs/1.514 GMACs. In addition, the measured inference time was 4.01±1.95 ms per 30-second epoch on an NVIDIA A100 GPU. This efficiency is consistent with the architectural design: depthwise separable convolutions limit parameter growth, while temporal compression reduces the sequence passed to the recurrent module from 500 raw samples to only 5 latent time steps. However, direct computational comparisons with previous methods should be interpreted cautiously because reported runtime values were obtained using different hardware platforms and experimental settings.

Taken together, the comparative results in Tables 4 and 5 indicate that the proposed approach achieves highly competitive performance across both SleepEDF datasets. On SleepEDF-20, it achieves the best reported values among the compared methods for accuracy, Kappa, macro-F1, sensitivity, and specificity. On SleepEDF-78, it achieves the best reported accuracy, macro-F1, sensitivity, and specificity among the compared models, while its Kappa is slightly below the best reported value. Importantly, the proposed framework also achieves the highest N1 F1-score among the compared single-channel approaches on both datasets. Therefore, the main advantage of the proposed method is not limited to a single global metric but is reflected in a favorable balance between overall performance, minority-stage recognition, computational compactness, and prediction reliability.

Despite these encouraging benchmark results, translation to routine clinical use requires additional investigation. Regarding the gap between the current application and real-time clinical deployment, the present study evaluates the proposed framework in an offline cross-validation setting. Nevertheless, the architecture is compatible with near-real-time inference because prediction is performed using short EEG segments and compact latent representations. Model training would be performed offline, whereas only the trained model would be required during clinical inference. To bridge the gap toward real-time clinical use, future work should integrate the framework with streaming EEG acquisition, online preprocessing, artifact rejection, device-specific calibration, prospective clinical validation, and deployment on clinically approved hardware or suitable edge-computing devices. Although the measured inference time demonstrates computational feasibility under the experimental hardware configuration, prospective evaluation on wearable or resource-constrained clinical hardware is required before real-time applicability can be established in practice.

Beyond these engineering requirements, population generalizability must also be carefully considered before routine clinical deployment. The proposed framework was developed and validated using the adult SleepEDF-20 and SleepEDF-78 datasets. Although the architecture demonstrates strong performance on these adult benchmark datasets, its applicability to pediatric populations remains to be established. Sleep architecture and EEG characteristics differ substantially between children and adults and undergo rapid developmental changes throughout childhood, potentially requiring different modeling strategies or additional model adaptation for pediatric sleep staging [44,45]. Therefore, validation on dedicated pediatric sleep datasets is necessary before extending the proposed framework to these populations. Similarly, patients with sleep disorders or neurological conditions associated with altered sleep architecture, such as epilepsy, may require additional validation and potential model adaptation before clinical application [46,47].

A further methodological consideration concerns the dependence of supervised sleep-staging systems on expert-annotated hypnograms. Although supervised approaches, including the proposed framework, can effectively learn from expert annotations, their performance depends on both the availability and the reliability of manual labels [48]. The annotation uncertainty discussed above for N1 represents a specific manifestation of this broader issue. More generally, expert-scored hypnograms should be regarded as a clinical reference standard rather than an absolute physiological ground truth, because sleep is a continuous and complex physiological process that is discretized into predefined stages for clinical scoring [49].

In this context, future research may benefit from investigating self-supervised and unsupervised learning approaches for automated sleep staging. Unsupervised methods may provide a more flexible framework by discovering intrinsic patterns directly from physiological signals without requiring extensive manual annotation [50]. However, defining clinically meaningful sleep states without expert guidance remains a major challenge. Previous studies have explored unsupervised and quasi-supervised strategies for automated sleep analysis, but their clinical interpretation and validation remain more challenging than those of conventional supervised approaches [51]. A promising future direction may therefore involve hybrid strategies that combine self-supervised representation learning, unsupervised pattern discovery, and expert knowledge to develop sleep-analysis systems that are more robust, data-efficient, and physiologically grounded.

Overall, the proposed framework provides an effective and computationally compact approach to automatic single-channel EEG sleep staging by combining multi-scale temporal feature extraction, temporal compression, hierarchical contextual modeling, and strategies for mitigating class imbalance. The ablation results provide empirical support for the complementary contributions of the major architectural components, while the confusion-matrix and spectral analyses show that the remaining errors largely follow physiologically plausible patterns. In addition, the segment-importance and calibration analyses provide complementary information about model behavior and prediction reliability. The proposed method achieves leading overall performance on most of the evaluated metrics and particularly strong performance for the challenging N1 stage across the two SleepEDF benchmarks. Nevertheless, prospective clinical validation, evaluation on more diverse populations, assessment on wearable or edge hardware, and further investigation of annotation uncertainty remain necessary before routine clinical deployment.

## Conclusion

As discussed earlier, data imbalance and limited receptive-field modeling remain among the most critical challenges in automatic sleep staging. In addition, developing lightweight models that can operate efficiently in real-time settings is an important practical requirement. In this work, we proposed a novel and lightweight single-channel EEG-based architecture built upon an efficient multi-branch convolutional compressor and a hierarchical BiLSTM framework, combined with class-weighted loss functions and data augmentation strategies to address class imbalance. Using only a single EEG channel, the proposed model achieved an accuracy of 89.72% and a macro-average F1-score of 85.46% under 5-fold cross-validation on the SleepEDF-20 dataset. Notably, the model attained an F1-score of 61.7% for the challenging N1 stage, representing a substantial improvement over existing single-channel EEG-based approaches. Overall, the proposed method outperformed prior techniques, providing an effective and computationally efficient framework for automatic sleep staging using single-channel EEG. Future work may focus on further improving model reliability and adaptability in practical scenarios. Moreover, evaluating the approach on clinical datasets and wearable devices would provide deeper insight into its performance under real-world conditions.
